# QTL Analysis and CAPS Marker Development Linked with Russet in Pear (*Pyrus* spp.)

**DOI:** 10.3390/plants11233196

**Published:** 2022-11-22

**Authors:** Yumi Kim, Sewon Oh, Hyeondae Han, Daeil Kim

**Affiliations:** 1 Department of Horticulture, Chungbuk National University, Cheongju 28644, Republic of Korea; 2 Department of Horticultural Sciences, Institute of Food and Agricultural Science, Gulf Coast Research and Education Center, University of Florida, Wimauma, FL 33598, USA

**Keywords:** genetic factor, high-density genetic linkage map, marker-assisted selection, molecular breeding, russeting

## Abstract

The fruit skin types of pear (*Pyrus* spp.) are divided into russet, smooth, and intermediate. One of the important traits in pear breeding programs is russet on pear fruit skin because it affects the commercial value. In the present study, a high-density genetic linkage map of ‘Whangkeumbae’ (smooth) × ‘Minibae’ (russet) was constructed. In addition, quantitative trait loci (QTL) analysis was performed to identify russet related QTL and develop a cleaved amplified polymorphism sequence (CAPS) marker. Together with SNPs derived from Axiom Pear 70K Genotyping Array and genotyping-by-sequencing derived SNPs and SSRs generated in previous study, an integrated genetic linkage map of ‘Whangkeumbae’ × ‘Minibae’ was constructed. A total of 1263 markers were anchored in 17 linkage groups (LGs) with a total genetic distance of 1894.02 cM and an average marker density of 1.48 cM. The chromosome coverage of ‘Whangkeumbae’ × ‘Minibae’ map was improved because the SNPs derived from Axiom Pear 70K Genotyping Array were anchored. QTL analysis was performed using previous russet phenotype data evaluated with russet coverage and Hunter a. As a result of QTL analysis, russet coverage- and Hunter a-related QTLs were identified in LG8 of the ‘Whangkeumbae’ × ‘Minibae’ map, and SNPs located in the QTL region were heterozygous in the ‘Minibae’. Although the russet coverage- and Hunter a-related QTLs were commonly detected in LG8, the logarithm of odds values of SNPs in the QTL region were higher in QTL related to russet coverage than to Hunter a. The CAPS marker (CBp08ca01) was developed using an array SNP located in the russet coverage related QTL, and the genotype of CBp08ca01 showed a 1:1 ratio in ‘Whangkeumbae’ × ‘Minibae’ (χ^2^ = 0.65, *p* > 0.05). ‘Whangkeumbae’ and ‘Minibae’ were thought to have *rr* and *Rr* genotypes, respectively, and the genetic factors controlling the russet formation might be located in chromosome 8. The CBp08ca01 was able to select F_1_ individuals with less than 30% russet coverage. Thus, it will be a useful tool for marker-assisted selection in pears.

## 1. Introduction

Pear (*Pyrus* spp.), which belongs to the Maloideae subfamily in Rosaceae, is one of the most important temperate fruit trees worldwide [1]. *Pyrus* species have 17 basic chromosomes (x = 17). According to their geographical origin, pears are divided into Asian pears (*P*. *pyrifolia*, *P*. *bretschneideri*, and *P*. *ussuriensis*) and European pears (*P*. *communis*). The fruit skin types of Asian pear are classified into three types: russet, smooth, and intermediate [2].

The russet on fruit skin is caused by the accumulation of cork layer and the formation of microcracks [3]. The cork consists of suberin, lignin, cellulose, and hemicellulose [3,4]. Russet on fruit skin is an important horticultural trait in pear breeding because it can protect pear fruit against external stresses, including disease, insects, inappropriate weather conditions, and shipping [5]. However, spotted russet on pear fruit skin reduces commercial value. Since the russet of fruit skin occurs in an amorphous form, the russet coverage has been graded and evaluated visually [5,6,7].

Previous studies have found quantitative trait loci (QTL) associated with russet on pear fruit skin. Yamamoto et al. [5] detected QTL associated with russet formation in linkage group (LG) 8 of ‘Akiakari’ (*P*. *pyrifolia*) × ‘Taihaku’ (*P*. *pyrifolia*) and a SSR marker (Mdo.chr8.10) linked with the QTL. Genome-wide associated study (GWAS) was also performed to explore significant single nucleotide polymorphism (SNP) associated with russet using 84 Asian pear accessions and 16 full-sib families. The significant SNPs associated with russet were identified in LG8 [6]. Takeuchi et al. [7] identified haplotypes associated with *R*, a genetic factor of cork layer formation on pear skin, on chromosome 8. In addition, Jiang et al. [8] identified two QTLs and genes associated with russet formation on chromosome 8 in *P*. *pyrifolia*. A random amplified polymorphic DNA (RAPD) marker associated with russet formation has been developed using two *P*. *pyrifolia* populations, ‘Kousui’ × ‘Kinchaku’ and ‘Niitaka’ × ‘Chikusui’ [2]. The drawbacks of the RAPD marker are low reproducibility and dominant inheritance.

SNP array is a high-throughput genotyping method using fixed DNA sequence information [9]. Molecular markers developed using array SNPs are reliable because SNPs are located in certain chromosomes [10]. The cleaved amplified polymorphic sequence (CAPS) marker detects restriction fragment length polymorphism in digested PCR amplicons using a restriction enzyme [11]. CAPS markers have been developed based on array SNPs in various crops, such as wheat, radish, and barley [9,12,13].

‘Whangkuembae’ (*P*. *pyrifolia*) shows slight russet formation on fruit skin. On the other hand, ‘Minibae’ ((*P*. *pyrifolia* × *P*. *ussuriensis*) × *P*. *pyrifolia*) displays abundant russet formation on fruit skin. Therefore, ‘Whangkeumbae’ × ‘Minibae’ is suitable for the identification of genes or QTL related to russet formation. Previously, a genetic linkage map of ‘Whangkeumbae’ × ‘Minibae’ was constructed using GBS-SNPs and SSRs [14]. The genetic linkage map of ‘Whangkeumbae’ × ‘Minibae’, with a total genetic distance of 1511.1 cM and an average marker density of 4.4 cM, covered 67.5% of the pear genome [14]. However, a reduction in marker density and an increase in genome coverage for QTL analysis and development of molecular markers related to traits of interest are necessary. In addition, Kim et al. [15] evaluated russet in Hunter a by colorimeter and russet coverage by image analysis in the fruits of ’Whangkeumbae’ × ‘Minibae’. Positive and negative Hunter a values represent the red and green, respectively. Because the russet on pear fruit skin appears reddish brown, Hunter a was analyzed for russet phenotyping. F_1_ individuals having russet fruit skin had positive Hunter a. The distributions of Hunter a and russet coverage in ‘Whangkeumbae’ × ‘Minibae’ were diverse, but image analysis evaluated russet more accurately than Hunter a. 

The high-density genetic linkage map is a foundation for the identification of loci associated with traits of interest. Moreover, the importance of precise phenotyping for QTL analysis in plants has increased. Therefore, the present study was performed (1) to improve the genetic linkage map of ‘Whangkeumbae’ × ‘Minibae’ by anchoring array SNPs, (2) to detect russet-related QTL and compare the results of QTL analysis using previous phenotype data evaluated by Hunter a and russet coverage [15], and (3) to develop a CAPS marker linked to russet.

## 2. Results

### 2.1. Genetic Linkage Map of ‘Whangkeumbae’ × ‘Minibae’

The genetic linkage map ‘Whangkeumbae’ × ‘Minibae’ anchored 1263 markers, including 976 array SNPs, 355 GBS-SNPs, and 7 SSR markers (Table 1 and Figure S1). Seventeen LGs corresponded to the 17 chromosomes of pear. The genetic linkage map covered 1894.02 cM (about 86.8% of the pear genome), with an average marker density of 1.48 cM. The number of markers on LGs varied from 53 to 142 (LG 5 to LG 9), and genetic distance ranged from 67.01 to 177.97 cM (LG 6 to LG 9). Thirty-three SSRs, including a previously developed SSR marker associated with russet formation (Mdo.chr8.10), were not mapped due to discordance of the segregation ratio. 

### 2.2. QTL Associated with Russet

**Figure 1:**
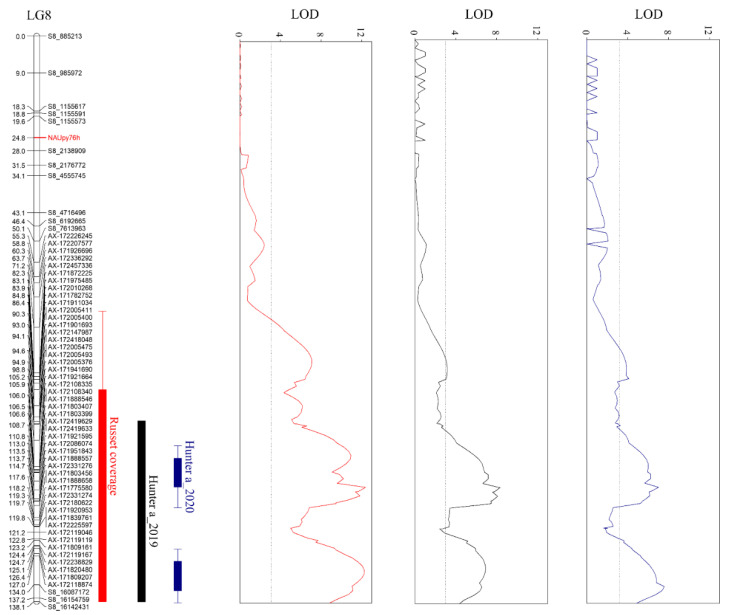
QTLs controlling russet formation on pear fruit skin located in linkage group (LG) 8 of ‘Whangkeumbae’ × ‘Minibae’. The left and right sides of the LG bar indicate genetic distance and anchored markers SNPs, respectively. Logarithm of odds (LOD) threshold is indicated by the dotted line. The three bars located on the right side of LG8 are QTL regions identified using different phenotyping methods. The QTLs and LOD graphs associated with russet coverage, Hunter a in 2019, and Hunter a in 2020 are shown in red, black, and blue, respectively.

QTLs associated with russet coverage and Hunter a were detected in LG8 of the ‘Whangkeumbae’ × ‘Minibae’ map. In the case of QTL associated with russet coverage, it was distributed from 71.2 to 138.0 cM, and logarithm of odds (LOD) values were 4.3 to 11.3 with an LOD threshold of 3.1. QTLs associated with Hunter a in 2019 were distributed 98.8 to 138.0 cM, and LOD values were 3.2 to 7.6. QTLs for Hunter a in 2020 were located from 82.3 to 139.0 cM, and LOD values were 3.2 to 7.5. The LOD thresholds of Hunter a in 2019 and 2020 were 3.0 and 3.2, respectively (Figure 1 and Table S1). The QTLs associated with russet coverage covered 10,088,795 to 16,154,759 bp in chromosome 8 of the ‘Dangshansuli’ (*P*. *bretschneideri*) genome. However, the QTLs for Hunter a ranged from 12,485,588 to 16,154,759 bp. The 47 significant SNPs (*p* < 0.0005) in common between russet coverage and Hunter a were anchored in the QTL region. These SNPs are heterozygous paternally (<nn × np>) and both parents (<hk × hk>). Among them, 35 SNPs have the genotype <nn × np>; the locus is heterozygous in ‘Minibae’.

### 2.3. CAPS Marker Discriminating Smooth and Russet Pears

**Figure 2:**
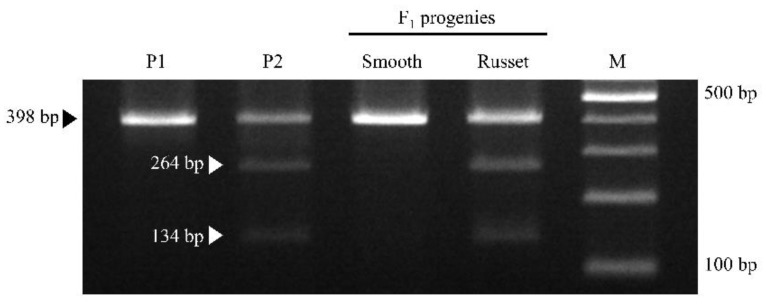
Application result of CBp08ca01 in ‘Whangkeumbae’ (P1), ‘Minibae’ (P2), smooth F_1_, and russet F_1_. The F_1_ DNA samples consisted of 20 individuals representing smooth and russet types. Black and white triangles indicate DNA fragments generated from smooth and russet pears, respectively. M, 100 bp ladder marker.

Sixteen CAPS markers were designed based on 47 SNPs located in QTL associated with russet. Among the 16 CAPS markers, only one CAPS marker (AX-172418048, LOD values 5.58) displayed segregation consistent with phenotype segregation in ‘Whangkeumbae’ × ‘Minibae’, and it was named CBp08ca01 (Table 2). The polymorphic SNP of CBp08ca01 was located at 11,010,613 bp in chromosome 8 of the pear reference genome. Using the primer pair of CBp08ca01, 398 bp amplicons were produced in ‘Whangkeumbae’, ‘Minibae’, and the two bulked F_1_ DNA samples. After the digestion treatment with *Rsa*I, 398 bp amplicon was generated in ‘Whangkeumbae’ and smooth F_1_ with the homozygous G allele. On the other hand, two DNA fragments of (134 and 264 bp) were generated in ‘Minibae’ and russet F_1_ with the R allele (A or G) (Figure 2). The genotype of the CAPS marker was separated by a 1:1 ratio (χ^2^ = 0.65). In addition, the CBp08ca01 could select F_1_ individuals with less than 30% russet coverage with a selectivity of ~70% selection rate (Table S2).

## 3. Discussion

The development of high-throughput screening technologies for genotyping and phenotyping has accelerated association studies, including QTL and GWAS analyses, in plants. One of the high-throughput genotyping technologies, GBS, was utilized to construct an integrated genetic linkage map of ‘Whangkeumbae’ × ‘Minibae’ [14]. GBS is a high-throughput genotyping method, but it could not produce fixed SNPs because its sequencing results are affected by restriction enzymes [16]. On the other hand, the SNP array has the advantage of producing fixed SNPs [17,18]. Thus, the genetic linkage map of ‘Whangkeumbae’ × ‘Minibae’ was newly constructed using anchoring array SNPs. Compared to the previous genetic linkage map of Han et al. [14], the integrated genetic linkage map of ‘Whangkeumbae’ × ‘Minibae’ (Table 2 and Figure S1) was improved in terms of total genetic distance and marker density. The total genetic distance was about 383 cM longer, and the marker density became 2.92 cM denser than in the previous map (genetic distance of 1152.1 cM with average marker density of 4.4 cM). Moreover, the number of array SNPs anchored in the genetic linkage map of ‘Whangkeumbae’ × ‘Minibae’ was ~2.7-fold higher than that of GBS-SNPs. The chromosome coverage of 17 LGs was about 20% higher than the previous genetic linkage map. Thus, the genomic resolution of the newly constructed genetic linkage map of ‘Whangkeumbae’ × ‘Minibae’ has been improved.

Although 4.2 times more SNPs were mapped in the ‘Whangkeumbae’ × ‘Minibae’ map than in the previous map [14], the number of mapped SSR markers was extremely decreased. In particular, the SSR Mdo.chr8.10 associated with loci controlling russet in pear [5] was not mapped because of segregation distortion. SSR covers relatively larger genomic regions than SNP. Therefore, the reason for the decrease in the number of SSRs was thought to be a tight linkage between SNPs [19].

Kim et al. [15] suggested that the evaluation of russet (russet coverage) and Hunter a displayed various phenotype distribution in ‘Whangkeumbae’ (smooth) × ‘Minibae’ (russet). This means that one or more genes could control russet formation in pear fruit skin. As a result of QTL analysis, both russet coverage and Hunter a related QTLs were detected in LG8 corresponding to chromosome 8 (Figure 1). However, approximately two-fold higher LOD values were observed in the QTL associated with russet coverage, than those related to Hunter a. An accurate phenotype is important for identifying QTLs [20], and the image analysis data expressed as russet coverage was a more accurate phenotyping method than Hunter a [15]. Thus, the QTL associated with russet coverage was considered reliable. 

Previous studies have reported russet-related QTLs located on chromosome 8. Yamamoto et al. [5] identified russet-related QTL in LG8 of ‘Akiakari’ (russet fruit skin) map, and SSRs developed at 551, 3952 bp and an unknown position on chromosome 8 of apple genome were linked to the QTL. Minamikawa et al. [6] and Jiang et al. [8] also found russet-related QTLs on chromosome 8. However, the physical locations of the SNPs located in the russet-related QTLs have been shown as scaffold positions on chromosome 8 of the *P*. *bretschneideri* genome [21]. Although it is difficult to compare the physical locations of the russet QTLs identified in this study (Figure 1) and previous studies [6,8], we could suggest that the russet-related QTL covers 10,088,795 to 16,154,759 bp of chromosome 8 of the *P. bretschneideri* genome [22].Thus, we suggested that the genetic factors controlling russet formation is located in 10,088,795 to 16,154,759 bp of chromosome 8, and this result could serve as a basis for dissecting genomic characteristics related to russet formation on pear fruit skin.

Among the significant SNPs (*p* < 0.0005) included in the QTL region, ~70% SNPs have the paternal heterozygous genotype of <nn × np> (Figure 2 and Table S1). Kikuchi [23] suggested that two genetic factors, namely the major dominant factor *R* and recessive factor *r*, control cork layer formation on pear fruit skin. Recently, Takeuchi et al. [7] confirmed the haplotypes associated with *R* on chromosome 8 and revealed that russet pears had dominant factors *RR* and *Rr*, while smooth pears had recessive factor *rr*. Based on these backgrounds, we thought that ‘Whangkeumbae’ (smooth) would have *rr* and that ‘Minibae’ (russet) would have *Rr* because the significant SNPs in QTL were associated with russet have the genotype <nn × np>. Theoretically, the F_1_ individuals of ‘Whangkeumbae’ × ‘Minibae’ must show a 1:1 segregation ratio of russet to smooth. However, the F_1_ individuals displayed various russet coverage [14]. Heterozygous *Rr* appeared to be affected by the environment, and *RR* was not [23]. Therefore, it was thought that F_1_ individuals of ‘Whangkeumbae’ (*rr*) × ‘Minibae’ (*Rr*) showed various russet coverage.

In the previous study, RAPD and SSR markers associated with russet were developed in pear [2,5,7]. However, RAPD marker has the weakness of low reproducibility. In addition, the availability of SSR markers linked with russet [2,5,7] have not been demonstrated in other pear populations. In the present study, the CAPS marker (CBp08ca01) was developed using the significant array SNP associated with russet formation and was applied in ‘Whangkeumbae’ × ‘Minibae’ (Table S2). The segregation ratio of CBp08ca01 genotype fitted the expected ratio of 1:1 (*p* > 0.05). Because *Rr* is affected by environmental factors, the CBp08ca01 could distinguish the russet at a 30% level. Therefore, the CAPS marker CBp08ca01 will be used to select individuals that are abundant or scarce in russet in the juvenile phase.

## 4. Materials and Methods

### 4.1. Plant Materials and DNA Extraction

‘Whangkeumbae’ (*P. pyrifolia*), ‘Minibae’ ((*P*. *pyrifoila* × *P*. *ussuriensis*) × *P*. *pyrifoila*), and 183 F_1_ individuals grown in the orchard of the Pear Research Station, National Institute of Horticultural and Herbal Science, Naju, Republic of Korea (35°01’27.9” N, 126°44’44.5” E) were used. Fresh young leaves of ‘Whangkeumbae’, ‘Minibae’, and their F_1_ individuals were collected and preserved at −70 ℃ until DNA extraction. Genomic DNA was extracted with DNeasy Plant Mini Kit (Qiagen, Hilden, Germany) according to the manufacturer’s instructions. The quality and quantity of DNA were measured using Denovix DS-11 spectrophotometry (Denovix, Wilmington, NC, USA).

### 4.2. Genotyping with Affymetrix Axiom^®^ Pear 70K Genotyping SNP Array

The Axiom^®^ 70K Pear SNP array developed by Montanari et al. [24] was applied to ‘Whangkeumbae’, ‘Minibae’, and 183 F_1_. Raw data were filtered to each step using Axiom Analysis Suit v4.0 software. The quality control (QC) threshold was as follows: dish quality control (DQC) ≥0.82, QC call rate ≥90%, percentage of passing samples ≥95%, average call rate for passing samples ≥98.5%. 

Axiom Analysis Suite software performing Best Practice Workflow was used to classify SNPs of Affymetrix Axiom^®^ Pear 70K Genotyping Array into six classes. SNPs were sorted into the six classes of: PolyHighResolution (PHR), NoMinorHomozygote (NMH), MonoHighResolution (MHR), OffTargetVariant (OTV), CallRateBelowThreshold (CRBT), and other. A total of 71,363 sorted SNPs were detected. SNPs with missing values of 10% or less were used.

### 4.3. Construction of Genetic Linkage Map

Linkage analysis was performed using the mapping software JoinMap 5. The genetic linkage map was constructed with regression mapping algorithm and Kosambi’s mapping function. The genotype data consist of filtered 8662 array SNPs, 5965 GBS-SNPs, and 40 SSRs. Among them, the genotype data of 5965 GBS-SNPs and 37 SSRs were obtained from the previous study of Han et al. [14]. Three SSRs (CH01c06, Hi20b03, and Mdo.chr8.10) associated with russet formation [4,25] were additionally genotyped. Genotype data were scored as CP code in JoinMap 5: heterozygous maternal genotype (<lm × ll>), heterozygous paternal genotype (<nn × np>), and heterozygous both parents (<ab × cd>, <hk × hk>, and <ef × eg>). LGs were established with LOD threshold of 16.0. The markers mapped in each LGs were aligned based on the chromosomal level reference genome of *P. bretschneideri* [22]. Genetic linkage map of ‘Whangkeumbae’ × ‘Minibae’ was visualized using MapChart 2.3.

### 4.4. QTL Analysis

QTL analysis was conducted using MapQTL 6.0. Genotype data of SNPs and SSRs mapped in the integrated map of ‘Whangkeumbae’ × ‘Minibae’ and russet phenotype data representing Hunter a and russet coverage [9] were loaded into MapQTL 6.0. Interval mapping was applied to find QTL region(s). LOD threshold for interval mapping was decided based on permutation test with 1000 replicates. QTL regions were indicated using MapChart 2.3.

### 4.5. Development of CAPS Marker Associated with Russet Formation

Restriction sites containing SNPs located in the QTL region were identified using NEBcutter [26]. Primer 3 was employed to design CAPS markers. Flanking sequences (601 bp) including SNPs were extracted and used for primer design.

Two bulked DNA samples were prepared comprising 10 smooth and 10 russet F_1_ individuals out of 127 F_1_ individuals for screening candidate CAPS marker. PCR mixture (60 µL) contained HS Prime Taq Premix (2×) (GENETBIO, Daejeon, Republic of Korea), 10 pmole·µL^−1^ forward and reverse primers, distilled water, and 10 ng·µL^−1^ DNA. PCR was performed using the T100^™^ Thermal Cycler (Bio-Rad, Hercules, CA, USA), under the following conditions: initial denaturation at 94 ℃ for 5 min, followed by 34 cycles of denaturation at 94 ℃ for 30 s, annealing at 60 ℃ for 30 s, and extension at 72 ℃ for 1 min with final extension at 72 ℃ for 5 min. The 60 µL PCR products were purified with *AccuPrep*^®^ PCR/Gel Purification Kit (Bioneer, Daejeon, Republic of Korea) according to the manufacturer’s instructions.

The purified PCR products were digested according to the protocol for each restriction enzyme. The digested PCR amplicon was observed using 2.5% agarose gel electrophoresis with Azure c150 gel documentation system (Azure Biosystems, Dublin, CA, USA).

## 5. Conclusions

The high-throughput genotyping using SNP array and accurate phenotyping using image analysis were able to identify loci associated with russet formation on pear fruit skin. In particular, we could infer the genetic factors related to russet in ‘Whangkeumbae’ (smooth, *rr*) and ‘Minibae’ (russet, *Rr*) by identifying QTL in LG8. The CBp08ca01, which discriminates *rr* and *Rr* genotypes at 30% russet coverage, will contribute to increased pear breeding efficiency.

## Figures and Tables

**Table 1:** The number of anchored markers, genetic distance, and marker density on the genetic linkage map of ‘Whangkeumbae’ × ‘Minibae’.

Linkage Group	No. of Array SNPs	No. of GBS-SNPs	No. of SSRs	No. of Total Markers	Genetic Distance (cM)	Average Interval between Markers (cM)	Linkage Group Coverage (%)
1	56	15	0	71	96.59	1.36	87.72
2	44	29	2	75	121.50	1.62	87.08
3	104	8	0	112	104.06	0.92	92.68
4	59	9	0	68	105.34	1.54	95.95
5	27	25	1	53	101.50	1.91	98.41
6	51	9	0	60	67.01	1.11	59.05
7	17	52	0	69	122.28	1.77	82.36
8	48	14	1	63	138.07	2.19	88.68
9	107	35	0	142	177.97	1.25	90.30
10	71	8	0	79	122.79	1.55	92.19
11	91	16	1	108	116.80	1.08	99.60
12	37	28	0	65	98.06	1.50	66.97
13	64	24	0	88	115.23	1.30	73.41
14	49	18	0	67	100.47	1.49	86.56
15	66	21	1	88	141.63	1.60	90.07
16	26	28	1	55	164.72	2.99	94.14
17	59	16	0	75	109.11	1.45	90.51
Total	976	355	7	1263	1894.02		
Avg.				78		1.48	86.80

**Table 2:** Designed candidate CAPS markers associated with russet formation on pear fruit skin.

Name	Map Position (cM)	SNP ID	Restriction Enzyme	Primer Sequences (5′−3′)	Expected Size (bp)	SNP
CBp08ca01	94.086	AX-172418048	*Rsa*I	F: GATGTGCGTGGAGATGATGT	398 or 134/264	A/G
				R: AAATGTGTGTCCTCCGATCA		

## Supplementary Materials

The following supporting information can be downloaded at: https://www.mdpi.com/article/10.3390/plants11233196/s1, Figure S1: Genetic linkage map of ‘Whangkeumbae’ × ‘Minibae’. The linkage groups (LGs) correspond to the pseudochromosome of the pear genome. Marker name and genetic distance (cM) are listed on the right and left sides of each LG, respectively. SNP markers are indicated in black color. SNP markers having prefixes S and AX are GBS-SNPs and array SNPs, respectively. SSR markers of pear and apple are shown in green and red colors, respectively. Table S1: Information of QTLs associated with Hunter a in the linkage group 8 of ‘Whangkeumbae’ × ‘Minibae’; Table S2: Application of CBp08ca01 in ‘Whangkeumbae’, ‘Minibae’ and their 127 F_1_ individuals.
