# Supplementary material for: QTL Analysis and CAPS Marker Development Linked with Russet in Pear (Pyrus spp.)
Source: Plants (Basel). 2022 Nov 22;11(23):3196. doi: 10.3390/plants11233196 (PMC9739592; doi:10.3390/plants11233196)
Supplement: Supplementary file 1 [file plants-11-03196-s001.zip › Supplementary Table S1.pdf]

Supplementary Table S1. Information of QTLs associated with Hunter a in the linkage group 8 of 'Whangkeumbae' × 'Minibae'.

Locus	Map position (cM)	Segregation type	Hunter a in 2019		Hunter a in 2020		Russet coverage	
			LOD[1]	%Expl.[2]	LOD	%Expl.	LOD	%Expl.
AX-172457336	71.19	<nn × np>	1.59	5.6	1.94	7.7	4.4	11.8
AX-171872225	82.34	<nn × np>	3.13	10.6	3.85	14.7	6.52	17
AX-171975485	83.09	<nn × np>	3.06	10.4	4.10	15.7	6.42	16.8
AX-172010268	83.94	<nn × np>	2.3	8.0	2.99	11.7	5.37	14.2
AX-171782752	84.82	<nn × np>	2.49	8.6	3.20	12.4	5.58	14.8
AX-171911034	86.35	<nn × np>	2.09	7.2	2.71	10.6	4.35	11.7
AX-172005411	90.25	<nn × np>	2.17	7.5	2.77	10.9	6.15	16.1
AX-172005400	90.27	<nn × np>	2.17	7.5	2.77	10.9	6.15	16.1
AX-171901693	93.01	<nn × np>	2.46	8.5	3.07	11.9	5.1	13.6
AX-172147987	94.07	<nn × np>	2.45	8.4	2.99	11.7	5.56	14.7
AX-172418048	94.08	<nn × np>	2.45	8.4	2.99	11.7	5.58	14.7
AX-172005475	94.57	<nn × np>	2.77	9.5	3.28	12.7	6.59	17.2
AX-172005493	94.60	<nn × np>	2.77	9.5	3.28	12.7	6.6	17.2
AX-172005376	94.89	<nn × np>	2.59	8.9	2.92	11.4	6.11	16
AX-171941690	98.83	<nn × np>	4.09	13.7	4.10	15.6	9.58	24
AX-171921664	105.23	<nn × np>	6.71	21.5	5.99	22.0	9.66	24.1
AX-172108335	105.94	<nn × np>	6.84	21.8	5.95	21.9	9.11	22.9
AX-172108340	105.95	<nn × np>	6.84	21.8	5.95	21.96	9.11	22.9
AX-171888546	106.00	<nn × np>	6.87	21.9	5.98	22.0	9.17	23.1
AX-171803407	106.54	<nn × np>	7.15	22.7	6.24	22.8	9.76	24.4
AX-171803399	106.61	<nn × np>	7.17	22.7	6.26	22.9	9.83	24.5
AX-172419629	108.65	<nn × np>	6.66	21.3	5.67	21.0	9.61	24
AX-172419633	108.71	<nn × np>	6.66	21.3	5.67	21.0	9.59	24
AX-171921595	110.83	<hk × hk>	7.60	23.9	6.10	22.3	11.39	27.8
AX-172086074	113.02	<nn × np>	7.43	23.4	5.48	20.3	10.34	25.6
AX-171951843	113.48	<nn × np>	7.48	23.6	5.33	19.8	9.47	23.7
AX-171888557	113.72	<nn × np>	7.30	23.1	5.18	19.3	9.49	23.8
AX-172331276	114.71	<hk × hk>	3.41	11.5	2.57	10.1	6.85	17.8
AX-171803456	117.57	<hk × hk>	3.27	11.1	2.15	8.5	6.05	15.9
AX-171888658	117.57	<hk × hk>	3.27	11.1	2.15	8.5	6.05	15.9
AX-171775580	118.18	<nn × np>	3.39	11.5	2.41	9.5	6.15	16.1
AX-172331274	119.269	<nn × np>	3.33	11.3	2.58	10.1	5.8	15.3
AX-172180622	119.665	<hk × hk>	2.80	9.6	1.63	6.5	5.11	13.6
AX-171920953	119.754	<hk × hk>	2.75	9.4	2.02	8.0	5.08	13.5
AX-172225597	119.831	<hk × hk>	2.40	8.3	1.77	7.1	5.03	13.4
AX-171839761	119.831	<hk × hk>	2.44	8.4	1.80	7.2	4.97	13.3
AX-172119046	121.212	<hk × hk>	3.34	11.3	2.21	8.8	5.66	14.9
AX-172119119	122.761	<hk × hk>	5.18	17.0	3.65	14.1	7.7	19.8
AX-171809161	123.161	<hk × hk>	5.03	16.6	3.61	13.9	7.48	19.3
AX-172119167	124.42	<nn × np>	5.83	18.9	4.25	16.2	8.97	22.6
AX-172238829	124.743	<nn × np>	5.96	19.3	4.39	16.7	9.33	23.4
AX-171820480	125.071	<hk × hk>	5.93	19.2	4.51	17.0	9.47	23.7

Supplementary Table S1. Continued.

Locus	Map position (cM)	Segregation type	Hunter a in 2019		Hunter a in 2020		Russet coverage	
			LOD[z]	%Expl.[y]	LOD	%Expl.	LOD	%Expl.
AX-171809207	126.405	<nn × np>	6.40	20.6	5.20	19.4	10.48	25.9
AX-172118874	127.041	<nn × np>	6.62	21.2	5.39	20.0	11.03	27.1
S8_16087172	133.983	<hk × hk>	6.56	21.0	7.59	27.0	11.05	27.1
S8_16154759	137.151	<nn × np>	4.81	15.9	5.59	20.7	9.57	24
S8_16142431	138.077	<hk × hk>	4.35	14.5	4.83	18.2	8.85	22.4

[1]Logarithm of odds.

[2]Percentage of phenotype variance explained by QTL.
