# Supplementary material for: QTL Analysis and CAPS Marker Development Linked with Russet in Pear (Pyrus spp.)
Source: Plants (Basel). 2022 Nov 22;11(23):3196. doi: 10.3390/plants11233196 (PMC9739592; doi:10.3390/plants11233196)
Supplement: Supplementary file 1 [file plants-11-03196-s001.zip › Supplementary Table S2.pdf]

Supplementary Table S2. Application of CBp08ca01 in 'Whangkuembae', 'Minibae', and their 127 F_1 individuals.

Individual	Product size (bp)[1]	Phenotype[2]	Individual	Product size (bp)	Phenotype
Whangkeumbae	**398**	S	14_110	**398**	S
Minibae	**264/134**	R	14_113	**264/134**	R
14_53	**264/134**	R	14_114	**398**	S
14_54	**264/134**	R	14_115	**264/134**	R
14_55	**264/134**	R	14_116	**264/134**	R
14_56	264/134	S	15_1	**264/134**	R
14_57	**264/134**	R	15-2	**398**	S
14_58	**264/134**	R	15_3	**398**	S
14_59	**398**	S	15_4	398	R
14_60	**264/134**	R	15_6	**264/134**	R
14-61	**264/134**	R	15_7	**264/134**	R
14_63	**264/134**	R	15_8	**398**	S
14_64	**264/134**	R	15_14	**264/134**	R
14_65	**398**	S	15_15	**398**	S
14_66	**398**	S	15_16	**264/134**	R
14_68	398	R	15_17	398	R
14_70	**264/134**	R	15_18	398	R
14_71	**398**	S	15_19	**398**	S
14_72	**264/134**	R	15_20	264/134	S
14_73	398	R	15_21	**264/134**	R
14_76	**264/134**	R	15_22	**398**	S
14_77	**398**	S	15_23	398	R
14_78	**398**	S	15_25	264/134	S
14_79	398	R	15_26	**264/134**	R
14_80	398	R	15_27	264/134	S
14_84	**398**	S	15_28	**264/134**	R
14_85	**398**	S	15_32	**264/134**	R
14_86	398	R	15_36	398	R
14_87	**264/134**	R	15_37	**264/134**	R
14_88	398	R	15_39	**398**	S
14_89	**264/134**	R	15_41	264/134	S
14_90	**264/134**	R	15_46	**264/134**	R
14_91	**264/134**	R	15_48	**264/134**	R
14_92	398	R	15_49	398	R
14_93	**264/134**	R	15_50	**398**	S
14_94	**264/134**	R	15_51	**398**	S
14_95	**264/134**	R	15_54	398	R
14_96	**264/134**	R	15_55	**398**	S
14_98	398	R	15_56	**264/134**	R
14_100	398	R	15_57	**264/134**	R
14_102	**398**	S	15_59	**264/134**	R
14_103	**264/134**	R	15_60	264/134	S
14-105	398	R	15_61	**264/134**	R
14_107	**264/134**	R	15_62	398	R
14_108	398	R	15_65	**264/134**	R
14_109	**264/134**	R	15_66	398	R

Individual	Product size (bp)[z]	Phenotype[y]	Individual	Product size (bp)	Phenotype
15_68	**264/134**	R	15_101	**398**	S
15_69	398	R	15_103	**398**	S
15_71	**398**	S	15_104	**264/134**	R
15_72	**264/134**	R	15_107	398	R
15_75	264/134	S	15_110	398	R
15_77	398	R	15_111	398	R
15_81	264/134	S	16_1	**398**	S
15_82	**398**	S	16_2	398	R
15_84	**398**	S	16_4	**264/134**	R
15_85	**398**	S	16_5	**398**	S
15_86	**398**	S	16_6	**398**	S
15_88	**398**	S	16_9	398	R
15_89	**398**	S	16_12	264/134	S
15_91	**398**	S	16_18	**398**	S
15_93	**264/134**	R	16_19	**264/134**	R
15_95	**398**	S	16_14	**398**	S
15_96	264/134	S	16_15	264/134	S
15_97	398	R	16_16	**264/134**	R
15_98	**264/134**	R			

[1]The product sizes corresponding to the phenotype were indicated in bold.

[2]S, Smooth; R, russet
